# Research on Power Allocation in Multiple-Beam Space Division Access Based on NOMA for Underwater Optical Communication

**DOI:** 10.3390/s23031746

**Published:** 2023-02-03

**Authors:** Yanlong Li, Syed Agha Hassnain Mohsan, Xiao Chen, Riffat Tehseen, Shuaixing Li, Jianzhao Wang

**Affiliations:** 1 Optical Communications Laboratory, Ocean College, Zhejiang University, Zheda Road 1, Zhoushan 316021, China; 2 Ocean Research Center of Zhoushan, Zhejiang University, Zhoushan 316021, China; 3 Key Laboratory of Cognitive Radio Information Processing of the Ministry of Education, Guilin University of Electronic Technology, Guilin 541004, China

**Keywords:** underwater optical communication, non-orthogonal multiple access, max–min rates, power allocation

## Abstract

To meet the transmission requirements of different users in a multiple-beam access system for underwater optical communication (UWOC), this paper proposes a novel multiple-beam space division multiple access (MB-SDMA) system by utilizing a directional radiation communication beam of the hemispherical LED arrays. The system’s access users in the different beams are divided into two categories: the users with a single beam and the users with multiple beams. We also propose a power allocation algorithm that guarantees the quality of service (QoS) for single beam and multiple beam access, especially the QoS for edge users, and fairness for all users. An optimization model of power distribution under the constraints of specific light-emitting diode (LED) emission power is established for two scenarios, which ensure the user QoS for edge users and the max–min fairness for fair users. Using the Karush–Kuhn–Tucker (KKT) condition and the bisection method, we obtain the optimal power allocation expression for the two types of users in the optimization model. Through simulation, we verify that the proposed user classification and power allocation method can ensure the fairness of fair users on the premise of ensuring the QoS of edge users. At the same time, we know that the number of users will affect the improvement of the minimum rate, and the throughput of the non-orthogonal multiple access (NOMA) system is greatly improved compared with the traditional orthogonal multiple access (OMA) systems.

## 1. Introduction

The clusters of air and ground unmanned mobile devices and autonomous underwater vehicle robots (AUVs) can quickly access the network and perform tasks through mutual perception and information interaction. Compared with the air and ground robots, the AUV has not yet realized the node cluster. One of the obstacles is that the traditional RF signal attenuates very quickly underwater [1], while underwater acoustic communication has a narrow bandwidth and considerable delay [2]. Thus, it is unsuitable for interacting with large amounts of data from dense underwater nodes. Therefore, an effective realization of underwater node sensing and networking based on optical communication has become one of the most rapidly developing research fields of the underwater internet of things (IoT) in recent years [3]. This has attracted extensive attention from academia, industry, and the military worldwide. Underwater optical networking can play an essential role in ocean exploration and development in the future.

To make dense nodes form a better network, the traditional RF communication is based on a fixed resource allocation by the orthogonal allocation of the time, frequency, and code domains of the system [4]. This makes users communicate independently on the resources and does not interfere with each other. The latest wireless optical code division multiple access (OCDMA) technology has also been studied and implemented [5]. However, the number of nodes that can be served by an orthogonal multiple access system at the same time cannot exceed the number of orthogonal resources, therefore, the system throughput is limited. In order to meet the diverse transmission needs of future intelligent network transmission, it is necessary to study the access resource allocation for VLC access systems [6]. Optical access is significantly different from the multiple-access technology of RF communication due to the limited radiation distance and angle range. Therefore, it is important to use its directional radiation to form a beam for multi-access indoor optical access.

In a traditional massive MIMO system, each antenna unit is equipped with an RF chain, and a large number of RF chains may lead to high energy consumption. Hybrid precoding has been proposed to reduce the number of RF links in millimeter wave communications [7]. In order to improve system capacity for the multi-user access scenario of underwater cluster networking, multiple light-emitting diodes (LEDs) antenna arrays are deployed at the transmitter [8]. In [9], a novel massive multiple-input multiple-output (MIMO) transmission system is proposed in the beam domain for optical wireless communications. The optical base station equipped with massive optical transmitters communicates with a number of user terminals (UTs) through a transmit lens, so the beam division multiple access (BDMA) system is established. By utilizing the proposed semispherical lens on Beehive Structure Receiver (BSR), we can achieve the significant diversity channel gain which is required to enable massive Multiple Input Multiple Output (MIMO) technology in any VLC system [10]. By utilizing the proposed semispherical lens on Beehive Structure Receiver (BSR), we can achieve the significant diversity channel gain which is required to enable the space division multiple access technology in any VLC system [11].

**Figure 1:**
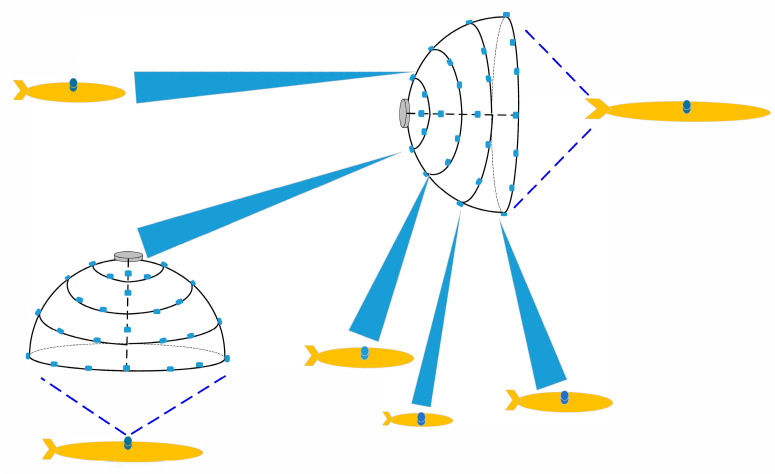
Underwater optical communication network based on the hemispherical LED array.

An underwater LED multiple-beam access system model is proposed based on the beam steering of the LEDs antenna array in this paper, as shown in Figure 1, in which the beam steering of LEDs antenna array technology is combined with the power domain (non-orthogonal multiple access) NOMA technology in the space division multiple access (SDMA) system. The novel multiple-beam space division access (MB-SDMA) system is that the NOMA technology is combined with multiple-beam to solve the problems existing in the traditional SDMA access system. Since the beam selection algorithm is used to convert the optical channel into the beamspace channel in the underwater optical network, there will be multiple optical links in the system. Therefore, multiple beams in the beamspace will suffer from inter-beam interference during demodulation [8,9,10]. So it is necessary to study the optical multiplexing method of visible light communication (VLC) in the beamspace to reduce the inter-beam interference while reducing the optical links and improving the access capacity.

In order to solve the problem of network forming for underwater nodes, this paper proposes a multiple-beam space division multiple access (MB-SDMA) system by utilizing a directional radiation communication beam of the hemispherical LED array, which uses an incoherent synthesis system to perform simple power superposition of array beams to achieve beam control [11]. This system proposes a multiple beam selection strategy aiming at the maximum receiving beam energy for the different spatial positions.

Then, an optimization model is established to make the user nodes satisfy the fairness criterion, which the fairness criterion means the rates tend to be the same for all users [12]. The optimization model will optimize the minimum rate of all users and maximize the minimum rate of all user nodes under the constraint of total transmit power. In [13], the description of edge users and fair users is given that the edge users are far away from the access node, and fair users are the user nodes that need to reach the same rate and QoS. Furthermore, fairness and system efficiency are usually contradictory objectives [14]. Thus, the non-convex model is solved using the bisection method and the Karush–Kuhn–Tucker (KKT) condition [15]. Finally, the multiple-beam access system proposes the power allocation algorithm to ensure user fairness. The main contributions of this paper are as follows:

- This paper proposes a new multiple-beam access system model with a hemispherical LED antenna array. We propose an antenna selection strategy for each user node, which scans all beams on the hemispherical LED array of the access node and selects the access beam based on the channel gain maximization. Finally, multiple space division beams are determined according to the relative position of the access nodes;
- A power allocation algorithm that guarantees the quality of service (QoS) for single-beam and multiple-beam NOMA access systems, especially the QoS for edge users and fairness for fair users, is proposed. This paper studies a power allocation algorithm considering both QoS and max–min fairness in a single-beam underwater visible light communication (UVLC) system;
- Considering the intra-beam and inter-beam interference for the multiple-beam access system, a fairness-oriented power allocation algorithm under the constraint of the total power of all users is proposed. We establish a model to maximize the minimum rate of all access users. Additionally, the bisection method and the KKT condition are used to solve the non-convex model.

## 2. Related Work

### 2.1. MB-SDMA and NOMA

In a traditional MIMO-VLC system, the capacity of the system is limited by the number of LED arrays at the transmitter [8]. However, increasing the LED arrays will bring problems such as increased hardware overhead and interference between beams. To meet the requirements of system throughput and spectral efficiency, 5G mobile communication proposes NOMA technologies. To use high spectral efficiency, low transmission latency, and no signaling interactive access, researchers systematically analyzed the ideas and advantages of NOMA technologies. They proposed the concept of power domain NOMA to improve spectral efficiency and system capacity [8]. Meanwhile, the analysis proved that MIMO-NOMA was superior to MIMO-OMA in channel and ergodic capacity [16]. Based on the research of NOMA and beam steering technologies, the power domain NOMA technologies are combined with multiple beams in the MB-SDMA system to solve the problems in the VLC access system. The power domain NOMA technologies perform power multiplexing at the transmitter and allocate power according to the channel gain of each user. Additionally, successive interference cancellation (SIC) is used to demodulate users according to the order of signal power. The design of the power allocation directly affects the demodulation performance of users [17]. In [18], to guarantee the QoS of users, researchers define a rate as a threshold rate for each user and propose a new power allocation strategy to maximize the achievable sum rate [19], in which the sum rate is the sum of the rates of all users. A power allocation strategy for VLC in NOMA systems is analyzed to maximize the system sum rate under the constraints of QoS, power consumption, and LED operating range (LOR) [20]. Considering the joint power allocation and antenna selection (J-PA-AS) problem of the downlink NOMA network cluster, there are studies on antenna selection for each user cluster and different transmitted power [21]. A power allocation strategy that maximizes the system sum rate under the multi-user QoS guarantee was proposed [22,23,24]. However, the multiple-beam NOMA still lacks detailed investigation.

### 2.2. QoS Optimization of Multiple-Beam NOMA

The sum rate of the dynamic and fixed power allocation algorithms in the downlink NOMA system is compared [25]. Simulation results show that dynamic fair power allocation can achieve a higher sum rate. Still, this dynamic fair power allocation does not take into account the QoS of edge users. A quasi-orthogonal space-time block code (Q-OSTBC) combined with NOMA is proposed to achieve better multi-path diversity under the total power constraints and QoS constraints [26], and it improves the system throughput. However, the user’s fairness is still not considered. In [27], the authors take energy efficiency, rate, and QoS into consideration and propose a method to jointly optimize system channel rate and optimal power allocation. It performs better in all aspects than existing schemes, but it does not consider optimizing the power distribution ratio of edge users. A SIC-free NOMA scheme based on constellation division coding is proposed to reduce the error propagation in VLC systems [28]. However, the SIC-free method does not meet the demodulation requirements of power domain NOMA at the receiver. Many researchers study optimal power allocation for a set of parallel channels when the transmitter has limited knowledge of the Channel State Information (CSI) [29]. Many researchers evaluate a cooperative diversity technique whereby a source broadcasts some data to a destination with the assistance of multiple relay nodes with Amplify-and-Forward (AF) protocols. By taking allocating power into account, it can increase the total capacity of the AF system [30], but the user’s fairness is not considered. For a multiuser Orthogonal Frequency Division Multiplexing (OFDM) based Cognitive Radio Networks (CRNs), a robust power allocation algorithm is designed to achieve the total transmit power minimization of Secondary Users (SUs) while the effect of uncertainties is suppressed effectively [31]. An algorithm to optimize the power allocation by minimizing the transmission completion time in energy-harvesting wireless relay networks is proposed [32]. Simulation results verify that the proposed algorithm can minimize the transmission completion time of the data transmission. Many studies exploited the solution of the maximal Signal-to-leakage ratio (maximal-SLR) for each user to find the minimum transmit power [33]. Still, it does not take into account the user’s QoS constraints.

Considering the shortcomings of the existing NOMA power allocation strategy, this paper first considers ensuring the transmission rate of edge users. At the same time, we consider the fairness of the second type of users and carry out power allocation to maximize the minimum rate. We finally established a power distribution model with a QoS guarantee and satisfied max–min fairness. For this non-convex model, we solved it with KKT conditions and the bisection method.

Most of the above research only considers the maximization of the sum rate of the system. In this case, the strong users will be allocated more communication power by the system, so the fairness of the rate of weak users cannot be guaranteed. At the same time, it is found that most of the power allocation algorithm research is based on a single NOMA user cluster without considering the problem of user access in multiple beams. So we will discuss the power allocation algorithm for multiple-beam NOMA systems under single and multiple-beam access situations.

## 3. System Model

**Figure 2:**
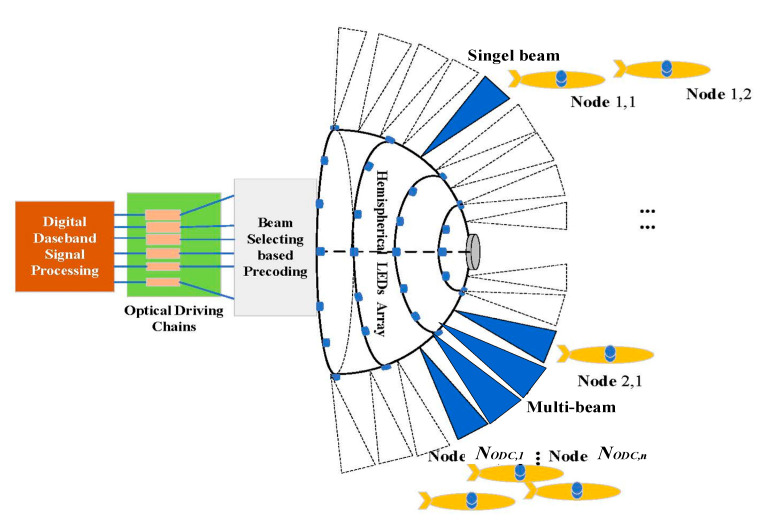
Schematic diagram of the hemispherical multiple-beam space division multiple access transmission.

As shown in Figure 1, we propose a hemispherical UVLC communication system. Our discussion is divided into a single beam and multiple beams, as shown in Figure 2, which is the multiple-beam NOMA access system. We deploy multiple LEDs in the direction of longitude and latitude on the hemispherical structure; when we only light a single LED for communication according to the user’s distribution position, it is a single beam access system, and when we light many LEDs for communication according to the user’s distribution position, it is multiple beam access system. In practice, the hemispherical LED antenna array on the AUV serving as the BS node activates the LEDs in each direction according to the latitude and longitude directions first. The access node, as a user, receives the optical signals at different positions in turn and stores the signal strength of the LEDs at different positions secondly. Finally, the LED beam with the biggest channel gain is selected as the access beam. Meanwhile, other LEDs are closed, and a single or multiple-beam access system is formed. The optical driving chains of the beam are converted to a beam space channel through beam selecting-based precoding. Then, based on the beam space channel, the BS can select some beams to serve all users, significantly reducing the number of optical driving chains, and each beam uses NOMA to serve multiple AUV nodes.

The system in this paper considers the case of communication in seawater. The composition of seawater is complex, including chlorophyll, suspended particles, and various organic matter, which have the effect of absorption and scattering of light in the propagation of light in the seawater. These two optical properties contribute to the power attenuation of light transmission in seawater. Therefore, according to Beer’s exponential decay model, the channel loss can be expressed as: $L_{ch}=\exp(-c(\lambda )d)$, where c(λ) is the total attenuation coefficient of seawater. Therefore, the channel gain $h_{ij}$ of the UVLC system can be expressed as:

(1) $$h_{ij}=\eta_{t}\eta_{r}A_{eff}\frac{(m+1)\cos^{m}\left(\phi_{ij}\right)}{2\pi }L_{ch},$$

where $\eta_{t}$ and $\eta_{r}$ represent the emission efficiency of the LED at the transmitter and the receiving efficiency of the photodetector at the receiver, respectively. $A_{eff}$ represents the effective receiving area of the photodetector, and *m* represents the Lambertian order, $m=-\ln(2)/\ln(\cos(\phi_{1/2}))$, where $\phi_{1/2}$ is the half-power half-angle of the LED, $\phi_{ij}$ represents the irradiance angle when the light of the *i*th LED is received by the *j*th detector, $L_{ch}$ is the total attenuation coefficient of seawater.

When calculating the channel gain, the ZEMAX software is used to obtain the transmitted power of the LED and the received power of the detector, so the DC gain of the underwater optical channel can be calculated as:

(2) $$h_{ij}=\frac{P_{r}^{j}}{P_{t}^{i}}\eta_{t}\eta_{r}L_{ch},$$

where $P_{r}^{j}$ is the optical power received by the *j*th detector, $P_{t}^{i}$ is the emitted optical power of the *i*th LED.

**Figure 3:**
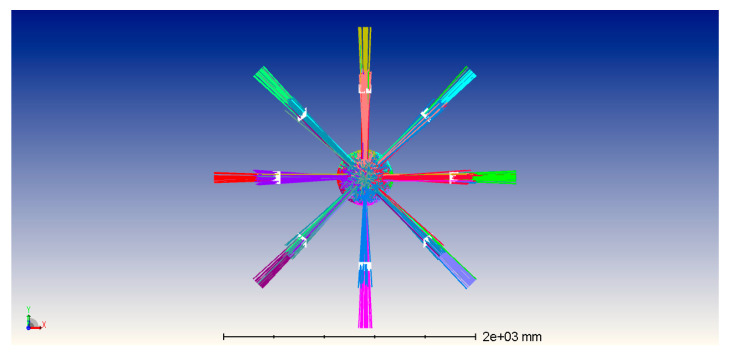
Transmitted beams by the hemispherical LED array.

As shown in Figure 3, we place 16 LEDs on the transmitter. LEDs are evenly distributed on the hemisphere according to the latitude and longitude direction, and the distance of the communication link is set to 10 m. Assuming that the transmitter and receiver are aligned, each detector forms a corresponding light spot on the receiving surface. As shown in Figure 3, the light spot was transmitted by an LED on its corresponding detector. We obtain the channel gains for users with different locations. Next, we discuss the selection of beams and power allocation for single-beam and multiple-beam NOMA systems.

Adopting a beam-space channel model can convert the traditional physical space channel model to the angle domain by using only a partially negligible performance loss. The transmitted multiple beams are considered in beam space, i.e., in the angular domain. Moreover, it can be seen that the channel matrix in the beam space is sparse, which is the basis for implementing the beam selection algorithm. Therefore, it is possible to take advantage of the sparsity of the channel matrix by selecting only a small part of the beams to complete the communication without compromising the overall system. To realize beam selection, precoding is introduced to choose which beams will finally be used. The following describes selecting and reducing the optical drive link by performing zero-forcing (ZF) precoding, thereby reducing power consumption and hardware complexity [34].

The maximum magnitude selection algorithm [35] is used in our single-beam and multiple-beam access system. The basic idea of the maximum amplitude selection algorithm is to select the beam with the largest amplitude of the channel matrix. To make the users choose the beam with the largest amplitude, we define the beam set as follows:

(3) $$\begin{array}{ccc} M^{\left(n\right)} & = & \left\{l\in \Gamma \left(N_{\mathrm{ODC}\;}\right):{\left|h_{l}^{n}\right|}^{2}\geq \xi^{\left(n\right)}\underset{l}{\max}{\left|h_{l}^{n}\right|}^{2}\right\},\\ M & = & \underset{n=1,\cdots ,\Omega_{l}}{\cup }M^{\left(n\right)} \end{array}$$

where l represents the ordinal number of the transmitted optical beam, $N_{ODC}$ represents optical driving chains, n represents the number of the nodes in the corresponding beam, and $\Omega_{l}$ represents the set of users in the *l*th beam. Among them, $h_{l}^{n}$ is the channel gain of the *l*th beam and the *n*th user. $M^{\left(n\right)}$ is the selection set for the *l*th user. $\xi^{\left(n\right)}\in [0,1]$ is the selection threshold, and different numbers of main beams can be selected by adjusting this value. In order to make each user select a closer beam so as reduce inter-beam interference, $\xi^{\left(n\right)}$ must be an independent value for each user. The transmitter uses the above formula to confirm the beam sequence number, which plays the most important role in each user’s signal receiving.

### 3.1. The Power Allocation Model for Single-Beam NOMA Access System

Firstly, we discuss the case of serving multiple users within a single beam. To improve the capacity of the single beam access system, a NOMA technique is introduced in resource allocation, and the system can adjust QoS for different users by power allocation. In the UVLC-NOMA system, we perform power allocation according to the channel conditions of different users. Less power is allocated to users with good channel conditions, and more power is allocated to users with poor channel conditions. The signals of multiple users are linearly superimposed and transmitted through power multiplexing by the LEDs at the transmitter, and the transmitted signal can be expressed as:

(4) $$x=\sum_{m=1}^{N}\sqrt{P_{m}}s_{m},$$

Here $s_{m}$ represents the signal of the *m*th user as it is a single beam access system, $P_{m}$ represents the power allocated to the *m*th user, *N* is the number of all users.

The signal received by the *m*th user within the beam range can be expressed as:

(5) $$y_{m}=h_{m}x+v_{m},$$

where $h_{m}$ denotes the channel gain of the *m*th user, m∈{1,2⋯,N}, and the channel gain is sorted in descending order, that is, $|h_{1}{|}^{2}\geq |h_{2}{|}^{2}\geq \ldots \geq |h_{N}{|}^{2}>0$, and $v_{m}\sim CN\left(0,\sigma^{2}\right)$ represents the thermal noise.

According to Shannon’s theorem [22], the information transmission rate of the *m*th user can be expressed as:

(6) $$R_{m}=\log_{2}\left(1+\frac{P_{m}{\left|h_{m}\right|}^{2}}{\sum_{k=1}^{m-1}P_{k}{\left|h_{m}\right|}^{2}+v_{m}^{2}}\right),$$

To meet the service requirements of different users, the users in the single beam are divided into two categories. The first category of users is edge users $m_{1}\in \{1,2,\cdots ,N^{’}\}$, where we allocate power to edge users first to satisfy QoS. The second type of users is fair users $m_{2}=\{N^{’}+1,N^{’}+2,\cdots N\}$, where we allocate all the remaining power to the second class of fair users to maximize the minimum rate. For $K_{1}$ and $K_{2}$, they satisfy $K_{1}\cap K_{2}=\emptyset$, $K_{1}\cup K_{2}=\{1,2,\cdots ,N\}$. The established optimization model is as follows:

(7) $$\begin{array}{c} \underset{\left\{p_{m}\right\}}{\max}\underset{m_{2}\in K_{2}}{\min}R_{m_{2}}\\ s.t.\;C_{1}:\sum_{m=1}^{N}P_{m}⩽P_{\max},\forall m\in K_{1}\cup K_{2}\\ C_{2}:R_{m_{1}}\geq R_{m_{1}}^{’},\forall m_{1}\in K_{1}\\ C_{3}:P_{m}\geq 0 \end{array}$$

Among them, $P_{m}$ is the power allocated by each user in the single beam, $P_{\max}$ is the total emission power of the LED, $R_{m_{1}}^{’}$ is the minimum rate which the first type of user needs to meet.

### 3.2. The Power Allocation Model for Multiple-Beam Access System

However, the number of optical drive links is limited, and each optical drive link with one beam accesses one user is not reasonable, so the number of system users is also limited. To break this limitation, we adopted multiple-beam NOMA technology; NOMA allows each beam to accommodate multiple users. Specifically, users served by the same beam are regarded as a NOMA group, multi-user signals are transmitted by superposition coding in the power domain of the beam, and SIC is performed in the beam for signal detection [36].

Therefore, the superimposed transmit signal at the AUV can be expressed as:

(8) $$x=\sum_{l=1}^{N_{\mathrm{ODC}\;}}x_{l}=\sum_{l=1}^{N_{\mathrm{ODC}\;}}\sum_{n=1}^{\Omega_{l}}\sqrt{P_{l,n}}S_{l,n},$$

where $x_{l}$ represents the superimposed signals within all beams, l represents the number of the nodes in the corresponding beam, $\Omega_{l}$ represents the set of users in the *l*th beam, and $N_{ODC}$ represents optical driving chains. $\sqrt{P_{l,n}}$ represents the power allocated to the corresponding users, and $S_{l,n}$ is the normalized transmitted signal.

Let l’ denote the beam serial number received by the node, then the received signal of the *n*th node in the l’ th beam can be expressed as:

(9) $$\begin{array}{cc} {\hat{y}}_{l^{\prime },n}= & {\hat{h}}_{l,n}x+z_{l,n}\\ = & {\hat{h}}_{l,n}\sum_{l=l’}^{N_{ODC}}\sum_{n=1}^{\Omega_{l}}\sqrt{P_{l,n}}S_{l,n}\\ + & {\hat{h}}_{l,n}\sum_{l\neq l^{\prime }}^{N_{\mathrm{ODC}}}\sum_{n=1}^{\Omega_{l}}\sqrt{P_{l,n}}s_{l,n}+z_{l,n} \end{array}$$

where ${\hat{h}}_{l,n}$ represents the corresponding beam space channel matrix after beam selection and $Z_{l,n}$ represents the channel noise $z_{l,n}\sim CN\left(0,\sigma^{2}\right)$. For users within the same beam, NOMA users are arranged in descending order of the beam space channel matrix gain, ${\left\|{\hat{h}}_{l,1}\right\|}^{2}>{\left\|{\hat{h}}_{l,2}\right\|}^{2}>\cdots >{\left\|{\hat{h}}_{l,n}\right\|}^{2}$. Therefore, in the *l*th beam, the channel quality of users is gradually reduced from the 1th user to the *n*th user.

To demodulate signals from multiple users, users within each beam use SIC to separate the signals. When we use SIC, the *i*th user (*i* < *n*) in the *l*th beam will first decode the user’s signal with a poor channel. The *i*th user will successively remove them from the signals which are received by the order n, n−1, …i until its signal can be detected. The residual signal (the residual signal after demodulation of the *i*th user) ${\hat{y}}_{l^{\prime },i(n)}$ is given by the following equation:

(10) $${\hat{y}}_{l^{\prime },i}={\hat{h}}_{l,i}^{}\sqrt{P_{l,n}}S_{l,n}+{\hat{h}}_{l,i}^{}\sum_{l=l^{\prime }}^{N_{\mathrm{ODC}\;}}\sum_{n=1}^{\Omega_{l}}\sqrt{P_{l,n}}S_{l,n}+{\hat{h}}_{l,i}^{}\sum_{l\neq l}^{N_{\mathrm{ODC}\;}}\sum_{n=1}^{\Omega_{l}}\sqrt{P_{l,n}}+z_{l,n}.$$

The *i*th user in the *l*th beam is interfered by the (*n −* 1)th users when decoding, and the signal-to-interference-noise ratio (SINR) $\gamma_{i,n}^{l}$ is given by the Equation (11):

(11) $$\gamma_{i,n}^{l}=\frac{{\left\|{\hat{h}}_{l,i}\right\|}_{2}^{2}P_{l,n}}{\phi_{i,n}^{l}+\sigma^{2}},$$

where $\phi_{i,n}^{l}$ is the sum of powers of the intra-beam and inter-beam interference, and $\sigma^{2}$ is the power of the thermal noise $Z_{l,n}$. $\phi_{i,n}^{l}$ is given by the following formula:

(12) $$\phi_{i,n}^{l}=\sum_{l=l^{’}}^{N_{\mathrm{ODC}\;}}\sum_{n=1,n\neq i}^{n}{\left\|{\hat{h}}_{l,i}\right\|}_{2}^{2}P_{l,n}+\sum_{l\neq l^{\prime }}^{N_{\mathrm{ODC}\;}}\sum_{n=1,n\neq i}^{n}{\left\|{\hat{h}}_{l,i}\right\|}_{2}^{2}P_{l,n},$$

combining Equation (12) and the Shannon equation, the corresponding achievable rate can be expressed as:

(13) $$R_{i,n}^{l}=\log_{2}\left(1+\gamma_{i,n}^{l}\right).$$

## 4. Optimization of Power Distribution Algorithm

For the system model proposed in Section 3, it is considered that the established optimization model is non-convex. To find the maximized minimal rate, we set the variable *t*, let $t=\underset{m_{2}\in K_{2}}{\min}R_{m_{2}}$; therefore, the power optimization model can be transformed as follows:

(14) $$\begin{array}{c} \underset{\left\{p_{m}\right\}}{\max}t\\ s.t.\;C_{1}:\sum_{m=1}^{N}P_{m}⩽P_{\max},\forall m\in K_{1}\cup K_{2}\\ C_{2}:R_{m_{1}}\geq R_{m_{1}}^{’},\forall m_{1}\in K_{1}\\ C_{3}:R_{m_{2}}\geq t,\forall m_{2}\in K_{2}\\ C_{4}:P_{m}\geq 0 \end{array}$$

In order to further solve the optimization model, the value of the variable *t* can be set to a fixed value $t_{0}$, so the optimization model can be further transformed as:

(15) $$\begin{array}{c} \underset{\left\{P_{m}\right\}}{\min}\sum_{m=1}^{N}P_{m}\\ s.t.\;C_{1}:R_{m_{1}}\geq R_{m_{1}}^{’},\forall m_{1}\in K_{1}\\ C_{2}:R_{m_{2}}\geq t_{0},\forall m_{2}\in K_{2}\\ C_{3}:P_{m}\geq 0 \end{array}$$

The KKT condition can specifically solve the above optimization model. Firstly, according to Equation (8), the constraints in the equation can be further transformed as follows:

(16) $$C_{1}:P_{m_{1}}{\left|h_{m_{1}}\right|}^{2}⩾\left(2^{R_{m_{1}}^{’}}-1\right)\left(\sum_{k=1}^{m_{1}-1}P_{k}{\left|h_{m_{1}}\right|}^{2}+\sigma_{n}^{2}\right),$$

(17) $$C_{2}:P_{m_{2}}{\left|h_{m_{2}}\right|}^{2}⩾\left(2^{t_{0}}-1\right)\left(\sum_{k=N^{’}+1}^{m_{2}-1}P_{k}{\left|h_{m_{2}}\right|}^{2}+\sigma_{n}^{2}\right),$$

Therefore, the Lagrangian function of Equation (8) can be expressed as follows:

(18) $$\begin{array}{ll} L(P,\lambda ,\mu ,\varphi )= & \sum_{m=1}^{N}P_{m}+\sum_{m_{1}\in K_{1}}\lambda_{m_{1}}\left(\left(2^{R_{m_{1}}^{’}}-1\right)\left(\sum_{k=1}^{m_{1}-1}P_{k}{\left|h_{m_{1}}\right|}^{2}+\sigma_{n}^{2}\right)-P_{m_{1}}{\left|h_{m_{1}}\right|}^{2}\right),\\ & +\sum_{m_{2}\in K_{2}}\mu_{m_{2}}\left(\left(2^{t_{0}}-1\right)\left(\sum_{k=N^{’}+1}^{m_{2}-1}P_{k}{\left|h_{m_{2}}\right|}^{2}+\sigma_{n}^{2}\right)-P_{m_{2}}{\left|h_{m_{2}}\right|}^{2}\right)-\sum_{m=1}^{N}\varphi_{m}P_{m} \end{array}$$

According to the KKT conditions, the above Lagrangian function can be solved. It is proved that the constraints $C_{1}$ and $C_{2}$ in the optimization model of Equation (8) are established [8]. It means that when the second type of user needs to meet the max–min fairness criteria, the rates of all NOMA users are similar, which proves that the algorithm makes the second type of user rate satisfy fairness. According to the KKT condition, the expression of user power allocation is obtained as follows:

(19) $$P_{m1}^{*}=(2^{R_{m_{1}}^{’}}-1)(\sum_{k=1}^{m_{1}-1}P_{k}+\frac{\sigma_{n}^{2}}{|h_{m1}{|}^{2}}),\forall m_{1}\in K_{1},$$

(20) $$P_{m2}^{*}=(2^{t_{0}}-1)(\sum_{k=N^{’}+1}^{m_{2}-1}P_{k}+\frac{\sigma_{n}^{2}}{|h_{m2}{|}^{2}}),\forall m_{2}\in K_{1},$$

To make the access rate of any node in multiple beams tend to be consistent, the minimum rate needs to be maximized. Additionally, it is necessary to ensure that the power allocated to each user is non-negative and that the total power of all users is less than the maximum transmission rate of $P_{\max}$. If there are m beams, the number of rows is m, and the corresponding objective function can be established as:

(21) $$\begin{array}{l} \max\min R_{i,n}^{l}\;\\ s.t.\;\sum_{l=l}^{N_{\mathrm{ODC}\;}}\sum_{n=1}^{\Omega_{l}}P_{l,n}\leq P_{\max}\\ P_{l,n}\geq 0 \end{array}$$

Therefore, to ensure fairness among user nodes, the minimum rate needs to be maximized. Suppose the minimum rate is $v=\min R_{i,n}^{l}$, if v is a certain constant value, it can be converted to [37]:

(22) $$\begin{array}{l} \min\sum_{l=1}^{N_{\mathrm{ODC}\;}}\sum_{n=1}^{\Omega_{l}}P_{l,n}\;\\ s.t.\;P_{l,n}\geq 0\\ R_{i,n}^{l}\geq v \end{array}$$

and $R_{i,n}^{l}\geq v$ is equivalent to $R_{i,n}^{l}=\log_{2}\left(1+\gamma_{i,n}^{l}\right)\geq v$:

(23) $${\left\|{\hat{h}}_{l,i}\right\|}_{2}^{2}P_{l,n}\geq \left(2^{v}-1\right)\left(\sum_{l=l^{\prime }}^{N_{\mathrm{ODC}\;}}\sum_{n=1,n\neq i}^{n}{\left\|{\hat{h}}_{l,i}\right\|}_{2}^{2}P_{l,n}\right.\left.+\sum_{l\neq l^{\prime }}^{N_{\mathrm{ODC}\;}}\sum_{n=1,n\neq i}^{n}{\left\|{\hat{h}}_{l,i}\right\|}_{2}^{2}P_{l,n}+z_{l,n}\right),$$

according to KKT conditions:

(24) $$L(P,\lambda ,\mu )=\sum_{l=1}^{N_{ODC}}\sum_{n=1}^{\Omega_{l}}P_{l,n}+\lambda \left(2^{v}-1\right)\left(\sum_{l=l}^{N_{\mathrm{ODC}\;}}\sum_{n=1,n\neq i}^{n}{\left\|{\hat{h}}_{l,i}\right\|}_{2}^{2}P_{l,n}\right.\left.+\sum_{l\neq l}^{N_{ODC}}\sum_{n=1}^{n}{\left\|{\hat{h}}_{l,i}\right\|}_{2}^{2}P_{l,n}+z_{l,n}\right)-\mu \sum_{l=1}^{N_{ODC}}\sum_{n=1}^{\Omega_{l}}P_{l,n}.$$

By solving Equations (25) and (26), it can be seen that the rates of all users are equal to the minimum rate v, that is, the rates of all NOMA users are equal to each other [36]. At this time, the maximum–minimum rate can be obtained by the dichotomy method, and the rates of different users can be obtained at the same time [38].

The following Algorithm 1 is designed to solve Equation (15).
**Algorithm 1****Input:**$P_{max}$,$\;\;h_{max}$,$\;\sigma_{n}^{2}$, $R_{m_{1}}$, m. **Output:**$t^{*}$, $P_{m_{2}}^{*}.$ 1: Set initial interval $t_{min}=0$, $t_{max}=\log_{2}\left(1+P_{max}h_{max}/\sigma_{n}^{2}\right)$ 2: Set $P_{max}-m*P_{m_{1}}^{*}$ obtained by $R_{m_{1}}$ and m. 3: **while**
$\left|t_{max}-t_{min}\right|>\varepsilon do$**do** 4:  Set $t=\left(t_{max}+t_{min}\right)/2$, bring the value of t into Equation (15) to obtain the power distribution coefficient $\left\{P_{m_{1}}^{’}\right\}.$ 5:  **if**
$\overset{N}{\underset{m_{2}=N+1}{\sum }}P_{m_{2}}\leq P_{max}-m*P_{m_{1}}^{*}$
**then**
6:   Set $t_{min}=t.$ 7:  **else** 8:   Set $t_{max}=t.$ 9:  **end if** 10: **end while** 11: Set $t^{*}=\left(t_{max}+t_{min}\right)$, $\left\{P_{m_{2}}^{*}\right\}=\left\{P_{m_{2}}\right\}$.

The following Algorithm 2 is designed to solve Equation (21) [39].
**Algorithm 2****Input**: $P_{max}$, ${\hat{h}}_{l,i}$, $z_{l,n}$, v, l, n. **Output:**$t^{*}$, $P_{l,n}^{*}.$ 1: Set initial interval $t_{min}=0$, $t_{max}=\log_{2}\left(1+\gamma_{i,n}^{l}\right)$ 2: Set $P_{max}-P_{l,n}$, obtained by l,n. 3: **while**$\left|t_{max}-t_{min}\right|>\varepsilon$**do** 4:  Set $t=\left(t_{max}+t_{min}\right)/2$, bring the value of t into Equation (22) to obtain the power distribution coefficient $\left\{P_{l,n}\right\}$. 5:  **if**
$(P_{\max}-\sum_{l=1}^{N_{\mathrm{ODC}\;}}\sum_{n=1}^{\Omega_{l}}P_{l,n}\;)>0$
**then** 6:   Set $t_{min}=t$. 7:  **else**
8:   Set $t_{max}=t$. 9:  **end if**
10: **end while** 11: Set $t^{*}=\left(t_{max}+t_{min}\right)$, $\left\{P_{l,n}^{*}\right\}=\left\{P_{l,n}\right\}$.

## 5. Experiment and Analysis

### 5.1. Simulation Settings and Analysis for a Single Beam System

In this section, we verify the performance of the proposed power allocation algorithm by simulation, and our simulation conditions are presented below. For both UVLC-NOMA and UVLC-OMA systems, we assume that a single LED is used at the transmitting end, which transmits the signal to users, and the number of users is set to 16 at the receiving end. For the channel gain calculation, we set the LED emission efficiency $\eta_{t}=0.1289$, the photodetector receiving efficiency $\eta_{r}=0.95$, the communication distance D=10 m, and the attenuation factor c=0.15. In this paper, the channel gains are set in descending order with different locations. To simulate the actual communication scenario, it is considered to classify users 15 and 16 as the first type of edge users, and users 1 to 14 as the second type of fairness users. When the QoS requirements of two edge users are the same, set $R_{m_{15,16}}^{’}=1$ bit/s/Hz. When the QoS requirements of two edge users are different, set $R_{m_{15}}^{’}=0.6$ bit/s/Hz, $R_{m_{16}}^{’}=0.6$ bit/s/Hz.

**Figure 4:**
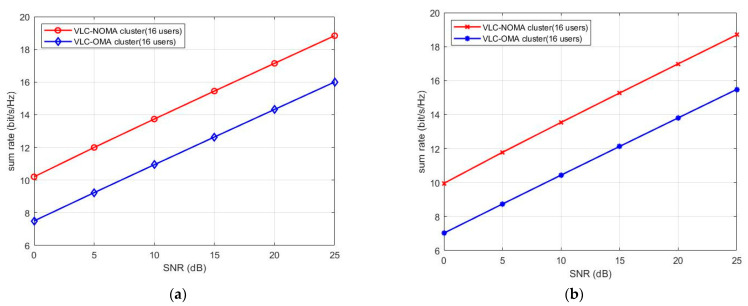
(**a**) Sum rate of NOMA and OMA under the same QoS. (**b**) Sum rate of NOMA and OMA under different QoS.

In particular, the conventional OMA system uses a time division multiple access (TDMA) scheme to serve numerous users. To maximize the minimum rate, the user is served with full power in each time slot. Figure 4a,b show the sum rates of UVLC-NOMA and UVLC-OMA at a different signal-to-noise ratio (SNR) under the same QoS and different QoS, respectively. Additionally, they show that NOMA improves the throughput by at least 30% compared to OMA. Moreover, with the improvement of SNR, the sum rate of NOMA is always higher than that of OMA.

**Figure 5:**
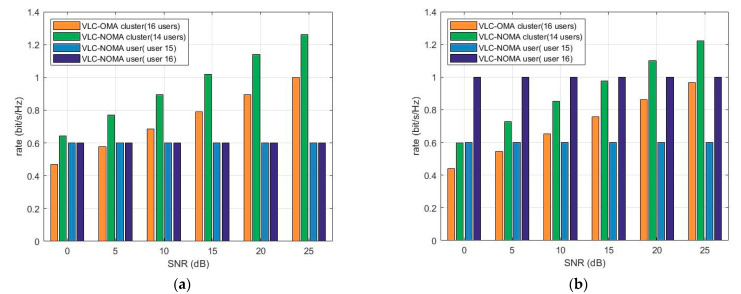
(**a**) Rates of different users under the same QoS. (**b**) Rates of different users under different QoS.

Figure 5a,b shows the rate of each user, where the histogram of the UVLC-NOMA cluster contains 14 s-class users for fairness. Under the same SNR after power allocation, the rates of all users in UVLC-NOMA are equal. The fair power optimization is performed on these 14 users, so all users in the second type of users have the same rate. The histograms of users 15 and 16 represent the rate of a single user, which is the minimum rate set by the algorithm under the QoS guarantee. When users 15 and 16 are under different QoS, the rate of user 16 is greater than the rate of user 15. It means the power allocation algorithm can optimize the power to make the edge user get the optimized QoS.

**Figure 6:**
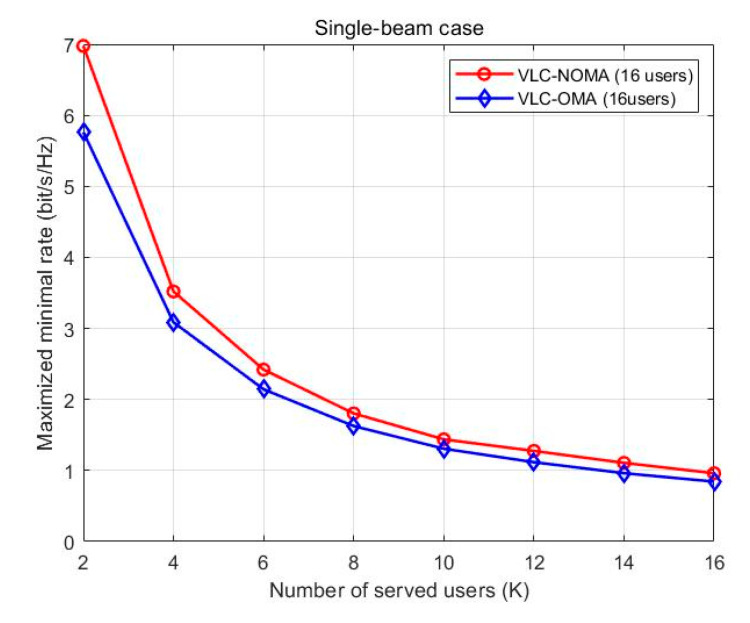
Maximized minimal rates of all users without QoS constraints.

Figure 6 shows that the maximized minimal rates vary with the number of users when there is no QoS constraint. In a single beam, a power allocation of max–min fairness is carried out to all users of UVLC-NOMA and UVLC-OMA systems. Figure 6 also shows that the maximized minimal rates of NOMA technology are greater than that of OMA technology in the UVLC access system over the entire SNR region. As shown in Figure 6, as the number of users increases, the maximized minimal rates of users drop sharply, but the maximized minimal rates of NOMA technology go down more slowly than the OMA technology in the UVLC access system as the number of users increases.

### 5.2. Simulation Settings and Analysis for Multiple-Beam System

**Figure 7:**
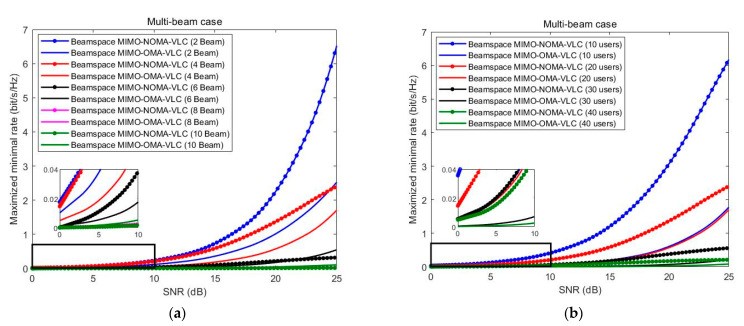
(**a**) The minimum rate varies with SNR in the case of multiple beams. (**b**) The minimum rate varies with SNR in a multi-user case.

As shown in Figure 7, in a multiple-beam NOMA system, the minimum rate analysis relative to the SNR for the different number of beams and a fixed number of users are given in multiple-beam systems; specifically, the system serves 20 users, and the number of beams is set to 2, 4, 6, 8, and 10. As shown in Figure 7a, the more beams there are, the lower the minimum rate of the system. In fact, as the number of beams increases, the interference between multiple beams will continue to increase, so the minimum rate of a system with more beams is smaller than a system with fewer beams. When the number of beams is less than 6, with the increase of SNR, the minimum rate of the multiple-beam NOMA and multiple-beam OMA systems both tend to increase, and the minimum rate of the NOMA system is significantly larger than the OMA system. When the number of beams is more than or equal to 6 and the number of beams is fixed, as the SNR increases, the growth slope of the minimum rate of beam space multiple-beam NOMA tends to decrease in the high SNR region. In contrast, the growth slope of the minimum rate of multiple-beam OMA still maintains an increasing trend in the high SNR region. However, when the number of beams is more than or equal to 10, the minimum rate of the NOMA system is smaller than the OMA system in the entire SNR range because the interference between beams is too large to make the NOMA system unable to improve its performance.

Therefore, in a multiple-beam NOMA system, the inter-beam interference of multiple-beam NOMA cannot be eliminated by digital precoding, which essentially limits the growth slope of its minimum rate and makes it limited in a high SNR region. On the contrary, since OMA adopts the TDMA scheme and ZF precoding method, multiple-beam OMA is free of inter-beam interference. Therefore, when the number of beams is within a certain range, as the SNR increases, the minimum rate of multiple-beam OMA will eventually exceed the minimum rate of multiple-beam NOMA when the SNR is sufficiently large.

Figure 7 depicts the minimum rate of the two systems when the number of serving users is set to 10, 20, 30, and 40, respectively, and the number of beams is set to 4. When the number of users served is small as the SNR increases, the minimum rates of the NOMA system and the OMA system both tend to increase, and the minimum rate of the multiple-beam NOMA is significantly larger than that of the multiple-beam OMA. The difference between the minimum rates is more significant in the high SNR region. However, since the inter-beam interference will become more and more severe with the increase in the number of served users, the minimum rate of multiple-beam NOMA is almost equal to the minimum rate of multiple-beam OMA. Since NOMA cannot reflect the advantages compared with OMA in the case of multiple users, when the number of beams is particularly large, the performance of NOMA is not as good as OMA in the entire SNR region, and it can be pointed out that the results are affected by inter-beam interference through theoretical analysis.

## 6. Conclusions

This paper proposes a multiple-beam access system model of the hemispherical LED array located in mobile carriers such as underwater AUVs to serve large numbers of users within a single beam and multiple beam. Considering the different service requirements of different users, different power allocation algorithms for single-beam NOMA and multiple-beam NOMA are proposed in this paper. Additionally, in a single-beam NOMA system, the users in a single beam are divided into two categories for the edge users and fair power allocation for the other users considering QoS constraints. By establishing the optimization model, we use the bisection method and the KKT condition to solve it, and we obtain the power distribution expression for the two types of users. By analyzing the simulation results, we find that the model guarantees the QoS of the first type of users and the fairness of the second type of users. Simultaneously, the maximized minimal rates drop sharply as the number of users increases. At the same time, the sum rate of NOMA is greater than that of OMA by comparing it with the OMA system.

In a multiple-beam NOMA system, by studying the influence of the number of beams on the minimum rate, we found that when the number of beams is within a certain range, the minimum rate of multiple-beam NOMA is always better than beam space OMA in the entire SNR area. When the number of beams is higher than the range, the rate of the multiple-beam NOMA system is lower than the multiple-beam OMA in the high SNR area. The minimum rate of multiple-beam NOMA is always better than multiple-beam OMA in the entire SNR range within a limited number of users. When the number of users is over a certain amount, the minimum rate of multiple-beam NOMA is almost equal to the minimum rate of multiple-beam OMA.

## Data Availability

The data used to support the findings of this study are available from the corresponding author upon request.

## References

[1] Hoeher P.A., Sticklus J., Harlakin A. (2021). Underwater Optical Wireless Communications in Swarm Robotics: A Tutorial. IEEE Commun. Surv. Tutor..

[2] Zeng Z., Fu S., Zhang H., Dong Y., Cheng J. (2016). A Survey of Underwater Optical Wireless Communications. IEEE Commun. Surv. Tutorials.

[3] Berlinger F., Gauci M., Nagpal R. (2021). Implicit coordination for 3D underwater collective behaviors in a fish-inspired robot swarm. Sci. Robot..

[4] Rahman M.R., Adedara K., Ashok A. (2020). Enabling multiple access in visible light communication using liquid crystal displays: A proof-of-concept study. Electronics.

[5] Akhoundi F., Salehi J.A., Tashakori A. (2015). Cellular Underwater Wireless Optical CDMA Network: Performance Analysis and Implementation Concepts. IEEE Trans. Commun..

[6] Zhou Z., Liu J., Yu J. (2021). A Survey of Underwater Multi-Robot Systems. IEEE/CAA J. Autom. Sin..

[7] Alkhateeb A., Leus G., Heath R.W. (2015). Limited Feedback Hybrid Precoding for Multi-User Millimeter Wave Systems. IEEE Trans. Wirel. Commun..

[8] Jiao R., Dai L., Wang W., Lyu F., Cheng N., Shen X. (2021). Max-min fairness for beamspace mimo-noma: From single-beam to multiple-beam. IEEE Trans. Wirel. Commun..

[9] Sun C., Gao X., Wang J., Ding Z., Xia X.-G. (2018). Beam Domain Massive MIMO for Optical Wireless Communications with Transmit Lens. IEEE Trans. Commun..

[10] Yaacobi A., Sun J., Moresco M., Leake G., Coolbaugh D., Watts M.R. (2014). Integrated phased array for wide-angle beam steering. Opt. Lett..

[11] Lang T., Li Z., Wang A., Chen G. Hemispherical Lens Featured Beehive Structure Receiver on Vehicular Massive MIMO Visible Light Communication System. Proceedings of the Internet of Vehicles—Safe and Intelligent Mobility.

[12] Bin-Obaid H.S., Trafalis T.B. (2020). Fairness in resource allocation: Foundation and applications. Proceedings of the International Conference on Network Analysis.

[13] Bychkov I., Kalyagin V.A., Pardalos P.M., Prokopyev O. (2020). Network Algorithms, Data Mining, and Applications.

[14] Bertsimas D., Farias V.F., Trichakis N. (2012). On the Efficiency-Fairness Trade-off. Manag. Sci..

[15] Timotheou S., Krikidis I. (2015). Fairness for Non-Orthogonal Multiple Access in 5G Systems. IEEE Signal Process. Lett..

[16] Li Y., Qiu H., Chen X., Fu J. (2020). A novel PAPR reduction algorithm for DCO-OFDM/OQAM system in underwater VLC. Opt. Commun..

[17] Ding Z., Liu Y., Choi J., Sun Q., Elkashlan M., Chih-Lin I., Poor H.V. (2017). Application of Non-Orthogonal Multiple Access in LTE and 5G Networks. IEEE Commun. Mag..

[18] Li Y., Qiu H., Chen X., Fu J., Musa M., Li X. (2019). Spatial correlation analysis of imaging MIMO for underwater visible light communication. Opt. Commun..

[19] Yang Y.S., Lee S.H., Chen W.C., Yang C.S., Huang Y.M., Hou T.W. (2022). Securing SCADA Energy Management System under DDos attacks using token verification approach. Appl. Sci..

[20] Higuchi K., Benjebbour A. (2015). Non-orthogonal Multiple Access (NOMA) with Successive Interference Cancellation for Future Radio Access. IEICE Trans. Commun..

[21] Shili M., Hajjaj M., Ammari M.L. (2021). Power allocation with QoS satisfaction in mmWave beamspace MIMO-NOMA. IET Commun..

[22] Yang F., Ji X., Liu X., Peng M. Power Allocation Optimization for NOMA based Visible Light Communications. Proceedings of the 2021 IEEE Wireless Communications and Networking Conference (WCNC).

[23] Miśkowicz M. (2021). Unfairness of Random Access with Collision Avoidance in Industrial Internet of Things Networks. Sensors.

[24] Su Y., Dong L., Yang Q. (2020). DCN-MAC: A Dynamic Channel Negotiation MAC Mechanism for Underwater Acoustic Sensor Networks. Sensors.

[25] Baidas M.W., AbdelGhaffar A.M., Alsusa E. Joint Power Allocation and Antenna Selection for Network Sum-Rate Maximization in Clustered Downlink NOMA Networks. Proceedings of the 2021 International Symposium on Networks, Computers and Communications (ISNCC).

[26] Yang Z., Xu W., Pan C., Pan Y., Chen M. (2017). On the Optimality of Power Allocation for NOMA Downlinks with Individual QoS Constraints. IEEE Commun. Lett..

[27] Wang H., Zhang Z., Chen X. Resource allocation for downlink joint space-time and power domain non-orthogonal multiple access. Proceedings of the 2017 9th International Conference on Wireless Communications and Signal Processing (WCSP).

[28] Zhu J., Wang J., Huang Y., He S., You X., Yang L. (2017). On Optimal Power Allocation for Downlink Non-Orthogonal Multiple Access Systems. IEEE J. Sel. Areas Commun..

[29] Chen C., Zhong W.-D., Yang H., Du P., Yang Y. (2018). Flexible-Rate SIC-Free NOMA for Downlink VLC Based on Constellation Partitioning Coding. IEEE Wirel. Commun. Lett..

[30] Razoumov L., Miller R.R. (2012). Power allocation under transmitter channel uncertainty and QoS constraints: Volumetric water-filling solution. J. Commun..

[31] Adam E.E.B., Yu L., Samb D. (2014). Performance Comparisons of Optimal Power Allocation over Nakagami-m and Rayleigh Fading Channels in Wireless Cooperative Systems. J. Commun..

[32] Xu Y., Chen Q., Tang L., Huang X. (2017). Robust power allocation for OFDM-based cognitive radio networks under signal-to-interference-plus-noise-ratio constraints. J. Commun..

[33] Xie Z., Zhu Q. (2017). Optimal Power Allocation Based on Transmission Completion Time Minimization for Energy Harvesting Relay Networks. J. Commun..

[34] Fadhil M., Abdullah N.F., Ismail M., Nordin R., Saif A., Al-Obaidi M. (2019). Power Allocation in Cooperative NOMA MU-MIMO Beamforming Based on Maximal SLR Precoding for 5G. J. Commun..

[35] Wu G., Jia D.-L., Lin W., Li S.-Q. (2010). Downlink Cooperative Transmission by Superposition Modulation: Performance Analysis and Power Allocation Strategy. J. Commun..

[36] Wang B., Dai L., Wang Z., Ge N., Zhou S. (2017). Spectrum and energy-efficient beamspace mimo-noma for millimeter-wave com-munications using lens antenna array. IEEE J. Sel. Areas Commun..

[37] Sayeed A., Brady J. Beamspace MIMO for high-dimensional multiuser communication at millimeter-wave frequencies. Proceedings of the 2013 IEEE Global Communications Conference (GLOBECOM).

[38] Sabril S., Jasman F., Hassan W.H.W., Mutalip Z.A., Mohd-Mokhtar R., Hassan Z. (2021). The Effect of Medium Inhomogeneity in Modeling Underwater Optical Wireless Communication. J. Commun..

[39] Hassan W.H.W., Sabril M.S., Jasman F., Idrus S.M. (2022). Experimental Study of Light Wave Propagation for Underwater Optical Wireless Communication (UOWC). J. Commun..

